# 

**Published:** 1875-03

**Authors:** 


### Contents of March Number.

### ORIGINAL DEPARTMENT.

ART. I.—Cerebro Spinal Anæmia and Convulsions. By W. W. Munson, M. D. .... 281
ART. II.—Abstract of the Proceedings of the Buffalo Medical Association. Feb. 2d and 9th, 1875, .... 295
ART. III.—Clinical Remarks upon Surgical Cases occurring in the Sisters of Charity Hospital. By Prof. J. F. Miner, M. D. Reported by W. W. Miner, M. D., .... 308

### MISCELLANEOUS.

The Therapeutic Value of the Iodide of Potassium .... 312

### EDITORIAL DEPARTMENT.

Meeting of the Alumni Association of the Buffalo Medical College .... 314
Items, .... 315
Obituary. Dr. Charles B. Coventry .... 317

BOOKS REVIEWED:

Croup in its Relations to Tracheotomy. By J. Solis Cohen, M. D. .... 317
Erysipelas and Childbed Fever. By Thomas C. Minor, M. D. 318
Surgical Emergencies. By Wm. P. Swain, M. D. .... 319
Outlines of the Science and Practice of Medicine. By Wm. Aitken, M. D., F. R. S., .... 319
Pulmonary Tuberculosis. By Addison P. Dutcher, M. D., .... 320
Books and Pamphlets Received .... 320

---

THE BUFFALO

